# Suicidal thoughts and behaviors among untreated illicit substance users: a population-based study

**DOI:** 10.1186/s12954-024-01015-9

**Published:** 2024-05-16

**Authors:** Ramin Shiraly, Seyed Amin Jazayeri, Asal Seifaei, Ali Khani Jeihooni, Mark D. Griffiths

**Affiliations:** 1 Community Medicine Department, School of Medicine, Shiraz University of Medical Sciences, Shiraz, Iran; 2 Student Research Committee, Shiraz University of Medical Sciences, Shiraz, Iran; 3 Nutrition Research Center, Public Health Department, Shiraz University of Medical Sciences, Shiraz, Iran; 4 International Gaming Research Unit, Psychology Department, Nottingham Trent University, 50 Shakespeare Street, Nottingham, NG1 4FQ UK

**Keywords:** Illicit substance use, Suicidal ideation, Attempted suicide, Heroin, Methamphetamine

## Abstract

**Objectives:**

Research regarding the contribution of specific psychoactive substances to suicidality has yielded equivocal results. The present study examined the prevalence and factors associated with suicidal thoughts and behaviors among a population-based sample of untreated illicit substance users.

**Methods:**

A total of 616 illicit substance users who were recruited from high-risk areas of Shiraz using snowball sampling participated in the study. Eligible participants were individuals aged 18 years and older who regularly used one illicit psychoactive substance (e.g., opioids, heroin, cannabinoids, stimulants, hallucinogens) for at least one year and who had received no treatment for their drug use during the past year. Data were collected regarding socio-demographic characteristics, mental history, and substance use habits. Data regarding suicidal thoughts and behaviors were assessed using the Beck Suicidal Ideation Scale (BSIS) and self-reports of previous suicide attempts. Multiple logistic regression analysis was used to identify independent variables associated with suicidality.

**Results:**

Among the participants, 23.6% reported having had suicidal thoughts during the past week and 6.7% reported having attempted suicide during the past year. Methamphetamine was reported as the primary substance of use among approximately half of the participants who attempted suicide during past year (49.2%). Multiple logistic regression analysis showed that current suicidal thoughts were independently associated with having no job, a history of mental health condition, previous suicidal attempts, concurrent use of more than one substance, and using methamphetamine and heroin as the primary substances. Suicidal thoughts were not associated with increased odds of regular opium and cannabis use.

**Conclusion:**

Both methamphetamine and heroin use are significantly associated with current suicidal thoughts. Evaluation of the risk of suicidality by physicians and mental health care professionals in both community and outpatient settings would be especially appropriate among those individuals using these psychoactive substances.

## Introduction

Suicide is a serious public health issue with more than 700,000 worldwide deaths annually with approximately 77% of cases from low- and middle-income countries [1]. There is a large body of evidence that substance use disorder is strongly associated with suicidal thoughts, attempts, and deaths [2–5]. According to a recent systematic review, 12% of deaths by suicide in low- and middle-income countries were associated with psychoactive substance use [6].

A global estimation of the annual prevalence of psychoactive substance use in 2015 suggested that the most common illicit substances used by the world adult population were cannabis (3.8%), amphetamines (0.77%), opioids (0.37%) and cocaine (0.35%) [7]. The present study was conducted in Iran, and at the country level, it has been estimated that 5% of Iranian general population, aged 15–64 years, regularly use at least one illicit substance during past 12 months [8]. A national study conducted by Iranian Ministry of Health in 2016 reported that 7.5% of Iranian adults occasionally used an illicit substance and 4.8% were regular illicit substance users. The most commonly used psychoactive substances reported by Iranian adults were opium, hashish, methamphetamine, heroin, hallucinogens and non-prescription methadone [9].

It is well-established that suicidality is associated with mental health illnesses, particularly mood disorders, psychotic disorders, and personality disorders [10]. Moreover, findings from the extant literature suggest that excessive substance use predicts subsequent suicidal ideation and behaviors after controlling for sociodemographic characteristics and psychiatric comorbidities [3, 11]. Studies have demonstrated that a significant proportion of substance-dependent individuals have pre-existing/co-existing psychiatric disorders, especially mood disorders and impulsive personality. Although in a large majority of cases, the mental health disorder precedes the substance disorder, one important theory to explain the high rates of co-occurrence of mental health disorders and substance use is that individuals use psychoactive substances to “self-medicate” and relieve their distressing psychiatric symptoms [12]. Substance use may drive suicidal desire through various mechanisms, such as increasing dysphoria, impulsivity, aggressiveness, and negatively impacting coping strategies [13].

One important issue that needs to be explored and understood is how specific psychoactive substances affect the relationship between substance use and suicide risk. The relationship between illicit drug use and suicidal thoughts/behaviors has been reported for specific substances such as heroin [14–16], methamphetamine [17, 18], cocaine [19], and cannabis [20–22]. It has been reported that heroin users are 14 times more likely than general population to die by suicide [9]. Suicidal ideation is a significant risk factor for future suicide attempts and death by suicide [23, 24]; it has been shown that current suicidal ideation is associated with 15–20% risk of suicidal behaviors within the next 12 months [25]. Additionally, suicidal thoughts have adverse effects on individuals’ mental health including decreased mental health-related quality of life and increased psychological distress [26, 27].

Although suicide risk is elevated among individuals with substance use disorders, the majority of substance-dependent individuals do not attempt suicide [28]. Therefore, identifying risk factors associated with suicidal thoughts and behaviors (including the influence of substances) among illicit substance users is of paramount importance. Some research studies that have investigated relationships between substance use and suicidal ideations and behaviors have mainly focused on legally used substances (i.e., alcohol and tobacco), and have therefore neglected illicit substances such as cannabis, opioids and stimulants [29]. Moreover, a lot of studies examining suicidality among illicit substance users have been conducted on individuals with a substance use disorder referred for treatment in addiction clinics and psychiatric services [e.g., 30, 31], and suicidality of untreated illicit substance users were less likely to be evaluated.

In the present study, a sample of Iranian illicit substance users who regularly used at least one psychoactive substance for at least one year and who had not received any formal treatment during that period were evaluated for current suicidal thoughts and past suicidal behaviors. It was hypothesized that the substance use pattern (including more frequent use of specific substances) would be associated with greater suicidality among illicit substance users.

## Methods

### Study setting and participants

The study was conducted in Shiraz, a city located in the Fras Province in South-western Iran and which has a population of approximately two million inhabitants. The study settings were inner-city neighborhoods of Shiraz with low socio-economic levels which are known as high risk for illicit substance use. Eligible participants were individuals aged 18 years and older, and who had regularly used one illicit psychoactive substance and who had not received any formal treatment for their drug use during the past year. The mean age of participants was 37.8 years (SD ± 13). Data were collected from July to August 2022. Complete anonymity and data confidentiality was guaranteed. The study was approved by the Shiraz University’s Ethics Committee (Ref: IR.sums.med.rec.1399.056) and was performed in accordance with the Declaration of Helsinki.

### Procedure

The data were collected through a face-to-face structured questionnaire by a team of trained researchers in public areas of high-risk neighborhoods in inner city of Shiraz. A total of 616 illicit substance users not currently undergoing treatment were recruited. After consulting with the Harm Reduction Unit of the Mental Health Department in Shiraz University of Medical Sciences, five major high risk disadvantaged neighborhoods in inner city of Shiraz were identified where illicit substance users gather and drug trafficking (buying and selling) takes place. The recruitment was initiated by research assistants after they had visited illicit substance users’ gatherings and invited eligible individuals to participate in the study and to complete the surveys in situ. Further cases were recruited through snowball sampling through the friendships and acquaintances of those initially recruited. Appointments were made to visit those who agreed to participate in the study. The survey was conducted through paper and pencil administration and took approximately 10 min for each participant to complete. Participants provided informed consent to participate prior to commencing the study.

### Measures

*Demographics.* Basic demographic data including age, gender, marital status, working status, and educational level were collected.

*History of diagnosed mental health condition.* Participants were asked if they have ever had any mental health condition diagnosed by a physician (including psychiatrists or primary care physicians) that necessitated regular physician visits and medication use (answered “yes” or “no”).

*Suicidal behaviors.* To evaluate suicidal behaviors, participants were asked (i) whether they had ever tried to die by suicide (lifetime suicide attempt) and (ii) whether they had tried to die by suicide within the past year (past-year suicide attempt) (both answered “yes” or “no”).

*Current suicidal ideation.* The Persian version of the Beck Suicidal Ideation Scale (BSIS) was used to assess current suicidal thoughts [32]. The scale comprises 19 items and assesses the presence of suicidal thoughts within the past week. Each item consists of three options which are rated on a three-point scale from 0 to 2. Total scores are computed by summing the items with a possible range of 0 to 38. Higher scores represent a higher intensity of suicidality. The first five items assess attitudes toward living and dying. Items 4 and 5 explicitly ask about passive or active suicidal desire and are used to screen for current suicidal ideations. Therefore, individuals who scored above 0 on either Item 4 or Item 5 were considered to have current suicidal ideation [33]. Psychometric properties of the Persian version of BSSI have been shown to be satisfactory [32]. Cronbach’s alpha in the present study was 0.968.

*Substance use habits.* To understand psychoactive substance use habits, participants were asked how often they used each substance in the past year. The response options were: (i) no use, (ii) occasional (less than daily) use, and (iii) regular (daily) use.

*Number of substances used concurrently.* Participants were asked how many psychoactive substances (including alcohol) they had used during the past year.

*Primary substance.* In the present study, the primary substance was defined as the most frequently used illicit substance during the past year reported by the participants. The respondents could select one illicit substance or substance type: opium, heroin, cannabis, methamphetamine, cocaine, hallucinogens, and non-prescription opioids.

### Statistical analysis

Descriptive statistics were used to calculate the demographic and other characteristics of the study participants. Demographic factors were calculated as frequencies and percentages for categorical variables, and means and standard deviations for numerical variables. Means were compared using independent *t*-tests. Categorical variables were analyzed using chi-square tests to determine the differences between groups. Multiple logistic regression was used to determine independent variables associated with current suicidal thoughts. Using backward stepwise binary logistic regression analysis, the presence of current suicidal thoughts (yes/no) was considered as the response variable, and seven predictors (age, marital status, job status, history of mental health condition, history of suicide attempt during past year, number of substances used and the most frequently used substance during past year) were selected for the final model. When building the final regression model, each of the variables listed above were evaluated in univariate models, and those with *p*-values < 0.20 were considered for inclusion in the backwards stepwise logistic regression. Assumptions of the models were tested using methods consistent with logistic regression (Hosmer and Lemeshow Goodness of Fit Test, and multicollinearity). SPSS version 28 (IBM, United States) was used for all statistical analysis and the significance level was *p* < 0.05.

## Results

The majority of the participants were males (81.2%) with less than 12 years of schooling (91.7%). Approximately half of the participants were married (49.3%), and half were single/divorced (50.7%). Approximately half had no job at the time of the study (49.8%). Approximately 6% reported a diagnosed mental health condition. The most frequently used illicit substances during past year reported by the participants were opium (48%), methamphetamine (27%), heroin (21%), and cannabis (4%). None of participants described other illicit substances (i.e., non-prescription opioids, cocaine, inhalants, and hallucinogens) as their most frequently used drug.

Almost half of participants reported that they had used at least one other substance in addition to the primary substance (46.8%). The most common substances co-used with their primary drugs were alcohol (50.7%), tramadol (16.8%), methadone (15.0%), opium (10.9%), methamphetamine (10.2%), heroin (9.1%), and cannabis (4.7%) (Table 1).

**Table 1:** Substance use habits of the participants (*n* = 616)

Psychoactive substance use characteristics	*N* (%)
Number of substances used concurrently	
One	328 (53.2)
Two	246 (40.0)
Three	31 (5.1)
≥ 4	11 (1.7)
Primary substance^*^	
Opium	293 (47.6)
Methamphetamine	166 (26.9)
Heroin	130 (21.1)
Cannabis	27 (4.4)
Substances co-used with the primary substance	
Alcohol	146 (50.7)
Tramadol	48 (16.8)
Methadone	43 (15.0)
Opium	31 (10.9)
Methamphetamine	29 (10.2)
Heroin	26 (9.1)
Cannabis	14 (4.7)

* The most frequently used substance during past year

Regarding suicidal thoughts and attempts, 23.6% of participants reported current suicidal thoughts, 10.4% had attempted suicide at least once during their lives, and 6.7% had attempted suicide during the past year. Among those who reported suicide attempt during the past year, about half (49.2%), reported methamphetamine and 21% reported heroin as their primary substance of use.

Table 2 shows the characteristics of adult illicit substance users who did and did not report suicidal thoughts during the past week. Those reporting suicidal thoughts were significantly more likely to (i) be single/divorced (62.2% vs. 43.1%; χ^2^(1) = 14.07, *p* < 0.001) (ii) be jobless (66.7% vs. 44.8%; χ^2^(1) = 19.41, *p* < 0.001), (iii) have a history of diagnosed psychiatric disorder (14.1% vs. 3.6%; χ^2^(1) = 19.52, *p* < 0.001), (iv) had history of attempted suicide (37.8% vs. 1.4%; χ^2^(1) = 142.01, *p* < 0.001), (v) have used more than one substance (39.3% vs. 23.8%; χ^2^(1) = 15.71, *p* < 0.001), and (vi) have used methamphetamine (43.7% vs. 21.4%; χ^2^(1) = 22.32, *p* < 0.001) or heroin (28.1% vs. 18.8%; χ^2^(1) = 17.67, *p* < 0.001) as their primary substance.

**Table 2:** Characteristics of study participants with or without suicidal thoughts (*n* = 616)

Characteristics	Without suicidal ideation (n = 471)	With suicidal ideation (n = 145)
	N (%)	N (%)
**Demographics**		
Age, mean (SD)	39.0 ± 13	36.6 ± 12.5
Gender		
*Male*	383 (81.4)	114 (78.5)
*Female*	88 (18.6)	31 (21.5)
Marital status		
*Married*	268 (56.9)	55 (37.8) ^***^
*Single/Divorced*	203 (43.1)	90 (62.2)
Work status		
*Have a job*	260 (55.2)	48 (33.3) ^***^
*Jobless*	211 (44.8)	97 (66.7)
Educational degrees		
*Less than 12 years of schooling*	437 (92.8)	134 (92.6)
*High school diploma or college*	34 (7.2)	11 (7.4)
**Mental history**		
History of mental disease		
*No*	454 (96.4)	125 (85.9) ^***^
*Yes*	17 (3.6)	20 (14.1)
History of suicide attempt		
*No*	464 (98.6)	90 (62.2) ^***^
*Yes*	7 (1.4)	55 (37.8)
**Substance use status**		
Number of illicit substances used		
1	359 (76.2)	88 (60.7) ^***^
2 or more	112 (23.8)	57 (39.3)
Primary substance		
*Methamphetamine*	101 (21.4)	63 (43.7) ^***^
*Heroin*	88 (18.8)	41 (28.1)
*Opium*	260 (55.2)	35 (24.4)
*Cannabis*	22 (4.6)	6 (3.8)

*p < 0.05, **p < 0.01, ***p < 0.001

Based on univariate regression analysis, seven predictors were selected as candidate variables for multivariate regression analysis (those with a *p*-value < 0.2). The final logistic regression model was fit and explained 39% of variation in the dependent variable (R^2^ = 0386). Based on the final step of the backward stepwise logistic regression analysis, current suicidal thoughts were associated with increased odds of not having a job (AOR:1.7, CI 95%:1.1–2.7, *p* = 0.019), having a history of diagnosed mental health condition (AOR:4.8, CI 95%:2.3–10.4, *p* < 0.001), having a history of suicide attempt (AOR:35.8, CI 95%:14.8–86.1, *p* < 0.001), using more than one illicit substance (AOR:1.7, CI 95%:1.1–2.7, *p* = 0.019) and using methamphetamine (AOR:4.4, CI 95%:1.6–12.2, *p* = 0.004) or heroin (AOR:3.1, CI 95%:1.1–8.8, *p* = 0.035) as their primary substances (Table 3).

**Table 3:** Results of univariate (unadjusted ORs) and backward stepwise logistic regression analysis (adjusted ORs) of factors associated with suicidal ideation among psychoactive substance users not currently undergoing treatment (*n* = 616)

Univariate model			Multivariate model	
**Factors**	Unadjusted OR (95% CI)	*p*-value	Adjusted OR (95% CI)^♯^	*p*-value
Age in years	0.98 (0.97–1.00)	0.077	0.99 (0.98–1.01)	0.902
Gender				
Male	1			
Female	1.20 (0.74–1.94)	0.454	-	-
Marital status				
Married	1		1	
Single/Divorced	2.17 (1.45–3.23)	< 0.001	0.69 (0.43–1.10)	0.123
Work status				
Have a job	1		1	
Jobless	2.46 (1.63–3.69)	< 0.001	1.70 (1.09–2.67)	0.019*
Educational level				
< 12 years of schooling	1		-	-
HS diploma or college	1.02 (0.48–2.16)	0.945		
History of mental health condition				
No	1		1	
Yes	4.36 (2.15–8.86)	< 0.001	4.89 (2.29–10.45)	< 0.001*
History of suicide attempt				
No	1		1	
Yes	34.21 (15.70–74.55)	< 0.001	35.78 (14.87–86.10)	< 0.001*
Number of substances used				
One	1		1	
2 or more	2.11 (1.40–3.20)	< 0.001	1.71 (1.09–2.68)	0.019*
Primary substance				
Opium	1		1	
Cannabis	1.22 (0.54–2.75)	0.629	1.48 (0.52–4.20)	0.46
Heroin	2.96 (1.29–6.83)	0.011	3.09 (1.08–8.84)	0.035^*^
Methamphetamine	4.52 (1.99–10.27)	< 0.001	4.41 (1.59–12.23)	0.004^*^

^*Statistically significant (*p*<0.05). ♯Adjusted ORs in the last step that the variable remained in the model^

## Discussion

The present study examined the prevalence and factors associated with suicidal thoughts and behaviors in a sample of illicit substance users not currently undergoing treatment from urban Shiraz community. The study found that using methamphetamine (a stimulant drug) was associated with highest odds of suicidal thoughts among illicit substance users. This finding is in line with previous research indicating serious consequences of use of amphetamine-type substances (ATSs) on mental health [34–36].

A recent systematic review and meta-analysis showed that using amphetamines was associated with higher odds of psychosis, depression, violence, and suicidality [34]. Also, a large French study that focused on the effect of psycho-stimulant substances on suicidal thoughts and behaviors among adolescents found that both cocaine and ATSs were associated with increased risk of suicidal ideation [37]. The same study also reported that the risk of lifetime suicidal attempts was higher among ATS users [37]. Additionally, the present study found a significant association between suicidal thoughts and increased odds of regular heroin use. This finding is consistent with results of studies which have shown that heroin use can be a strong predictor of future suicidal ideation, suicide attempts, and death by suicide [3, 14–16, 38, 39]. Given that the present study found increased odds of suicidal thoughts among heroin and methamphetamine users, the study’s hypothesis was confirmed.

It has previously been reported that using methamphetamines, heroin or a combination of opioids and stimulants are associated with increased risk of death. Excess mortality among methamphetamine/heroin users may be due to suicide or unintentional drug overdose or long-term medical conditions [40]. According to the World Health Organization (WHO), globally, 600,000 deaths were attributable to drug overdose in 2019, with approximately one quarter of those deaths caused by semisynthetic opioid overdose [41]. However, differentiating unintentional acute drug toxicity from intentional substance-related deaths is not easy in most cases, particularly in methamphetamine-related deaths [40]. Although the presence of suicidal thoughts among illicit substance users is a significant risk factor for future suicide attempts and deaths, non-substance-related methods of self-killing, such as hanging or death by firearms, are more frequent methods for suicide deaths among this population [42]. Furthermore, longitudinal studies have shown that most excess mortality among heroin and methamphetamine users are caused by long-term medical conditions [43, 44].

The present study found no significant increased odds of suicidal thoughts and behaviors with regular use of cannabis. This result is consistent with the findings of a large Swedish longitudinal study which concluded that cannabis use was unlikely to have a significant direct effect on the risk of suicide [45]. Also, a systematic review and meta-analysis by Borges et al. [46] concluded that the evidences on increased risk of suicidality by acute and chronic cannabis use are equivocal. Furthermore, a mediation analysis study by Buckner et al. found there was no direct effect of cannabis use on suicidality when psychological vulnerability factors were controlled for [47]. However, reports of associations between cannabis use and suicidal thoughts and behaviors have mixed findings. Although some studies have reported a positive association between cannabis use and suicidality [20–22], a systematic review including six related studies did not report any significant relationship [29].

The present study also found no significant association between using opium as the primary substance and current suicidal thoughts. Studies linking opium use to suicidality are scarce and most related literature has investigated opioids as a whole (including both natural opiates and semisynthetic and synthetic derivatives) and did not specifically explore associations with opium use. Consistent with the present study findings, a previous Iranian study found no association between opium use and suicidal ideations and behaviors [48]. However, some research suggests that although opiate overdose (especially when accompanied by alcohol consumption) and associated respiratory failure are implicated in many opioid use-related deaths, suicidality may play a significant role in opioid-related deaths [49].

Based on the results of the present study, suicidal thoughts appear to be quite common among illicit substance users (approximately one in four participants). The estimates of prevalence reported in the previous literature are also quite high. For example, a Spanish study reported that approximately one-third of illicit substance users referred to drug treatment and prevention facilities reported suicidal ideation in the last 12 months [50]. A recent meta-analysis of suicidal ideations among intravenous drug users reported a past-year pooled prevalence of 35% [51]. Another recent meta-analysis reported a pooled prevalence rate of 35% for suicide thoughts and 20% for suicide attempts in the past year among patients with substance use disorder. This review suggested that using cannabis, cocaine, and amphetamine were significantly associated with increased risk of suicidal attempts among individuals with SUD [52].

The present study estimated that 1 in 14 Iranian illicit substance users had attempted suicide during the past year and that 1 in 4 had current suicidal ideation. Given that these findings showed the potential relationship between using specific substances and suicidality, strategies to reduce suicidal thoughts and behaviors must be tailored accordingly. Given the relatively high prevalence of suicidal thoughts among untreated illicit substance users, strategies should be directed at effectively investigating the presence of suicidal thoughts and behaviors among this vulnerable population, particularly among those living in poor urban settings. Improving the availability of mental health facilities in underserved areas could help implementing this strategy.

It is globally estimated that only one in six individuals with substance use disorder receive treatment [53]. Individuals with substance use disorder may be reluctant to seek help for reasons such as fear of arrest and lack of trust [54]. Therefore, regular contact of social workers with these populations may encourage them to receive mental health services. Moreover, the risk of suicidality among illicit substance users varies based on the risk factors such as the number of substances used concurrently and the type of the primary substance used, therefore risk assessment of individuals who seek mental health services would help to identify those individuals who are in most need of mental health support. Development of specific risk factor-based screening tools/criteria for evaluation and stratification of suicidality among individuals with a substance use disorder may be a promising target for future research.

## Limitations

The present study had a number of limitations. The study comprised a medium sized non-probability sample of illicit substance users that may not be representative of all Iranian drug users. Moreover, neighbourhood context may influence individual behaviors and health outcomes. In the present study’s context (disadvantaged neighbourhoods), substance-related morbidity (e.g., psychological distress) and mortality may be disproportionately higher than privileged neighbourhoods. However, recruitment of individuals from high socio-economic backgrounds is arguably more difficult. Therefore, the results of the present study cannot necessarily generalized to illicit substance users from high socio-economic levels.

Another key limitation was that the study design was cross-sectional, relying on self-report data. Moreover, participants’ history of diagnosed psychiatric disorders was also assessed through self-report, rather than a diagnosis by a medical professional. Adverse childhood experiences which have been suggested as a contributing factor to adult suicidality [17, 55] were not evaluated. Also, current self-harming behaviors were not assessed (only current suicidal ideation). Cocaine and inhalants are rarely used by Iranian adults and no participant in the study reported non-prescription opioids and hallucinogens as the primary substance, therefore, their association with suicidal thoughts could not be evaluated. Since there is no universal consensus on a metric to quantify the frequency of substance use, in the present study, the primary substance was defined and assessed through self-report as the most frequently used illicit psychoactive substance during the past year to describe the main (principal) substance used by the participants. It should also be noted that the most frequently used substance does not necessarily mean the most preferred one, and factors such as the cost of the illicit substance may affect its choice for consumption. Considering the objectives of the study, tobacco smoking and alcohol use were not assessed as the primary substances, because both are legally used in most parts of the world.

Another study limitation is that the participants were not evaluated for fulfilment of substance use disorder by any screening criteria. Substance use exists on a continuum and not all substance use represents a diagnosable disorder. However, it has been argued that any level of substance use is unhealthy and can be associated with health risks and consequences even if not meeting criteria for a substance use disorder [56]. It has been shown that regular use of an illicit substance for at least one year may designate impaired control over substance use, representing mental and physical dependence [57].

## Conclusion

The present study indicated that suicidal thoughts are relatively common among Iranian illicit substance users, particularly among those with previous history of suicide attempts. With respect to the influence of substances, regular use of methamphetamine and heroin were associated with highest odds of suicidality compared to opium and cannabis. Evaluation of the risk of suicidality by physicians and mental health care professionals in both community and outpatient settings would be especially appropriate among those individuals using these psychoactive substances.

## Data Availability

The datasets generated and/or analyzed during the present study are not publicly available due to due to institutional regulations and privacy restrictions but are available from the corresponding author upon reasonable request.
